# Massachusetts Veterinary Association

**Published:** 1892-06

**Authors:** Austin Peters

**Affiliations:** Secretary


Meeting then adjourned.

(Signed) AUSTIN PETERS, *Secretary.*

---

*Massachusetts Veterinary Association.*—The annual meeting of the Massachusetts Veterinary Association was held at Young's Hotel, Boston, Wednesday evening, April 27th, 1892, at 5.30 o'clock. President L. H. Howard in the chair. Members present, Drs Bryden, Blackwood, Burr, Emerson, Harrington, Hitchings, Howard, Osgood, Peterson, Saunders, Winchester, Winslow, Hitchcock, Parker, and the Secretary. Honorary member, Dr. Stickney. Guests, Hon. Levi Stockbridge, Chairman of the Mass. Cattle Commission, and Dr. George H. Bailey, Veterinarian of the Maine Cattle Commission. Dr. W. L LaBaw was also present and elected to membership during the meeting.

The Records of the previous meeting were read and accepted. Unfinished business. The Secretary reports that Dr. Blackwood has attended more meetings for the year 1891 and 1892 than Dr. Marshall, and that he therefore is entitled to Dr. Lee's prize rasp.

Reports of Committees. Dr. Winchester as a member of the Committee of Arrangements for the annual meeting of the U. S. V. M. A. in Boston next September reports that there will probably be one hundred members present, outside of the Mass. Veterinary Association, and that perhaps it would be better to arrange for meeting in some small hall than at Young's Hotel. He also believes that any entertainment offered them by our State Association should be provided for by voluntary contribution, rather than by assessment.

Dr. Howard read a letter from Secretary Hoskins, of the U. S. V. M. A., in which he said that it was hard to give an exact estimate of the number to expect at the September meeting, but thought that one hundred might serve as a basis to figure upon. Admission of members. Dr. W. L. La Baw was elected a member by 11 unanimous votes.

Election of Officers. Dr. Winchester moved, that all officers be elected by ballot. Seconded by Dr. Winslow. Carried. Balloting then proceeded with Dr. Winslow acting as teller, an informal ballot being first taken, followed by a formal one, with the result of electing the following board of officers for the ensuing year.

President, L. H. Howard D. V. S., first Vice President, Alexander Burr, M. D. V., second Vice President, Edward C. Becket, M.D.V., Secretary and Treasurer,

Austin Peters, M. R. C. V. S. Executive Committee: F. H. Osgood, M. R. C. V.S., Daniel Emerson, M. D. V., Thos. Blackwood, D.V.S., Chas. Winslow, D. V. S., Wm. E. Peterson M. D. V.

The new Executive Committee reported favorably upon the credentials of Dr. F. B. Carlton, of Salem.

The Treasurer read his annual report, which was accepted upon the motion of Dr. Winchester.

The meeting then adjourned to the dining room where the annual dinner was partaken of, Dr. Bryden acting as toastmaster, a very jolly evening was passed, the members remaining until the *wee sma' hours.*

AUSTIN PETERS, *Secretary.*

